# Identification of prognostic splicing factors and exploration of their potential regulatory mechanisms in pancreatic adenocarcinoma

**DOI:** 10.7717/peerj.8380

**Published:** 2020-02-11

**Authors:** Min-hua Rong, Zhan-hui Zhu, Ying Guan, Mei-wei Li, Jia-shuo Zheng, Yue-qi Huang, Dan-ming Wei, Ying-mei Li, Xiao-ju Wu, Hui-ping Bu, Hui-liu Peng, Xiao-lin Wei, Guo-sheng Li, Ming-xuan Li, Ming-hui Chen, Su-ning Huang

**Affiliations:** 1 Affiliated Cancer Hospital, Guangxi Medical University, Research Department, Nanning, Guangxi Zhuang Autonomous Region, P.R. China; 2 Affiliated Cancer Hospital, Guangxi Medical University, Department of Radiotherapy, Nanning, Guangxi Zhuang Autonomous Region, P.R. China; 3 First Affiliated Hospital, Guangxi Medical University, Department of Pathology, Nanning, Guangxi Zhuang Autonomous Region, P.R. China

**Keywords:** Splicing factors, Pancreatic adenocarcinoma, RNA-sequencing, Overall survival

## Abstract

Pancreatic adenocarcinoma (PAAD), the most common subtype of pancreatic cancer, is a highly lethal disease. In this study, we integrated the expression profiles of splicing factors (SFs) of PAAD from RNA-sequencing data to provide a comprehensive view of the clinical significance of SFs. A prognostic index (PI) based on SFs was developed using the least absolute shrinkage and selection operator (LASSO) COX analysis. The PI exhibited excellent performance in predicting the status of overall survival of PAAD patients. We also used the percent spliced in (PSI) value obtained from SpliceSeq software to quantify different types of alternative splicing (AS). The prognostic value of AS events was explored using univariate COX and LASSO COX analyses; AS-based PIs were also proposed. The integration of prognosis-associated SFs and AS events suggested the potential regulatory mechanisms of splicing processes in PAAD. This study defined the markedly clinical significance of SFs and provided novel insight into their potential regulatory mechanisms.

## Introduction

Pancreatic cancer, the seventh most common cause of cancer-related death worldwide, is a highly lethal disease (Bray et al., 2018; Liang et al., 2018; Wang et al., 2018). According to epidemiological estimates in the United States, approximately 56,770 new pancreatic cancer cases were diagnosed and 45,750 people died from the disease in 2019 (Siegel, Miller & Jemal, 2019). Pancreatic adenocarcinoma (PAAD) is the predominant subtype of pancreatic cancer and remains a health priority (Chen et al., 2019; Kamisawa et al., 2016). Current treatments for PAAD include surgery, chemotherapy, radiation therapy, and palliative care; surgery is regarded as the only option for cure. However, most PAAD patients experience no symptoms in the early stages, which precludes surgical removal (Strobel et al., 2019). Hence, molecular biomarkers that can effectively monitor the onset and prognosis of PAAD are indispensable. In addition, the complex mechanisms underlying the development of PAAD remains poorly understood.

Splicing is an important process in vivo and is responsible for transcript diversity (Dvinge & Bradley, 2015; Kim et al., 2018). Splicing factors (SFs) are a powerful manipulator in modulating RNA processing and maintaining cellular homeostasis (Dvinge et al., 2016). More importantly, intricate splicing events are orchestrated by a limited number of SFs. Many studies have found links between the turbulences of SFs and the onset and progression of cancers (Cieply & Carstens, 2015; Shilo, Siegfried & Karni, 2015; Silipo, Gautrey & Tyson-Capper, 2015). In PAAD, SFs also exhibit potential effective functions in many ways. Adesso et al. (2013) found that silencing SRSF1, a member of the arginine/serine-rich splicing factor protein family, could facilitate apoptosis induced by gemcitabine via the MNK/eIF4E pathway (Adesso et al., 2013). This finding offers an alternative way to enhance gemcitabine efficiency in PAAD. However, studies with a focus on the functions of SFs in PAAD are still scarce. A comprehensive analysis to determine the clinical value of SFs in PAAD is urgently needed.

Here, we systematically analyzed the clinical significance of SFs in PAAD and provided clinically practicable molecular biomarkers. More importantly, a prognostic index (PI) based on the expression profiles of SFs was proposed, which offers excellent survival prediction. Moreover, we also explored the clinical significance of alternative splicing (AS) events. The PI based on AS events also demonstrated a satisfactory prognosis prediction performance. In addition, the SF-AS regulatory network also provides novel insight into the molecular function of SFs in PAAD.

## Methods

### Data acquisition

A catalog of 404 SF genes was obtained from a previous study (Seiler et al., 2018). The fragments per kilobase of transcript per million mapped reads (FPKM) data of PAAD patients were downloaded from the Cancer Genome Atlas (TCGA, https://cancergenome.nih.gov/) database using the TCGAbiolinks R software package (Colaprico et al., 2016). The corresponding clinical annotation had also been downloaded and extracted from the TCGA database. Gene name annotation was performed using an ensemble database (GRCh38.95). Next, the FPKM expression data were quantified to “transcripts per million” (TPM) data and normalized to the log2 (TPM+1) data type. Then, normalized TPM data was used for subsequent analysis.

### Survival analysis

The R package survival outputs were used for univariate COX analysis of selected prognosis-associated SFs. To obtain more accurate results, only PAAD patients with an overall survival (OS) greater than 90 days were included in the survival analysis. Then, we further conducted gene ontology (GO) and Kyoto Encyclopedia of Genes and Genomes (KEGG) pathway functional enrichment analysis to reveal the potential molecular functions of prognosis-related SFs. The GO analysis mainly includes biological processes (BPs), cellular components (CCs), and molecular function (MF). The gene functional enrichment analysis was conducted using the “clusterProfiler” package in R software (Yu et al., 2012).

### Survival-associated alternative splicing events

SFs performed their molecular function mainly by regulating the AS events process (Papasaikas & Valcárcel, 2016). We further systematically analyzed the prognostic value of alterations in AS events in PAAD and the associations between SFs and AS events. Transcript and splicing event details of cross-tumors of TCGA RNA-seq data were downloaded from the TCGA SpliceSeq database (https://bioinformatics.mdanderson.org/TCGASpliceSeq/) (Gao et al., 2019; Lin et al., 2019; Lin et al., 2018; Ryan et al., 2016; Zhang et al., 2018). The SpliceSeq database quantified the seven AS events, including Alternate Acceptor Site (AA), Alternate Donor Site (AD), Alternate Promoter (AP), Alternate Terminator (AT), Exon Skip (ES), Mutually Exclusive Exons (ME), and Retained Intron (RI), by calculating a percent-splice-in (PSI) value. The PSIs ranged from 0–1. A PSI value of an ES event of 0.8 indicates that the exon is contained in approximately 80% of the transcripts in the sample. We used splice event filters according to the following conditions: (1) Percentage of samples with a PSI > 75% and (2) a minimum PSI standard deviation > 0.1. The missing value was filled using the k-Nearest Neighbor (KNN) method. The KNN was conducted with the Impute package in R software. Next, we integrated the PSI values of AS events and the survival data of PAAD and conducted a univariate COX analysis to identify prognosis-associated AS events. AS events with a *P*-value <  0.005 were identified as prognosis-associated AS events.

### Construction of a PI

To develop a PI based on the expression profiles of SFs genes, a least absolute shrinkage and selection operator (LASSO) was conducted. Any SFs genes with *P*-values < 0.005 were identified as most the significant prognosis-related genes. Then, the selected most significant prognosis-related SFs were further screened and confirmed by the LASSO regression. The classifier was trained using 10-fold cross-validation to determine the optimal parameter configuration. The PI was established with the following formula: PI = expression level of SF 1 * *β*1 + expression of SF 2 * *β*2 + …expression of SF *n*∗ *β n*. We generated a risk score for each patient based on the PI. Then, PAAD patients were placed into groups of two according to the median value of PI (Qin et al., 2019).Furthermore, we used another gene expression dataset that were publicly available and reported clinical outcome information to be used as validation cohort. Gene expression matrix of pancreatic tumors patients in GSE62452 dataset was downloaded from the Gene Expression Omnibus (https://www.ncbi.nlm.nih.gov/geo/).

Similarly, the top 20 AS values that were closely related to the prognosis (except the number of ME <20) were subjected to a LASSO COX analysis to develop a PI based on AA, AD, AP, AT, ES, ME, and RI, respectively. Then, a final PI was generated by submitting the top 20 AS events for a LASSO COX analysis. The time-dependent incident dynamic ROCs with area under the curve (AUC) values were calculated to estimate the performance of each model (Blanche, Dartigues & Jacqmin-Gadda, 2013).

### SF-AS regulatory network

To construct an SF-AS regulatory network and learn more about the PI we proposed, we analyzed the relationships between SFs genes included in the PI and OS associated AS events. Co-expression relationships were identified by Pearson correlation analysis, and the threshold was set to correlation coefficient *r* > |0.6| with a *P*-value <0.05.

## Results

### Identification of prognosis-associated SFs

**Figure 1:**
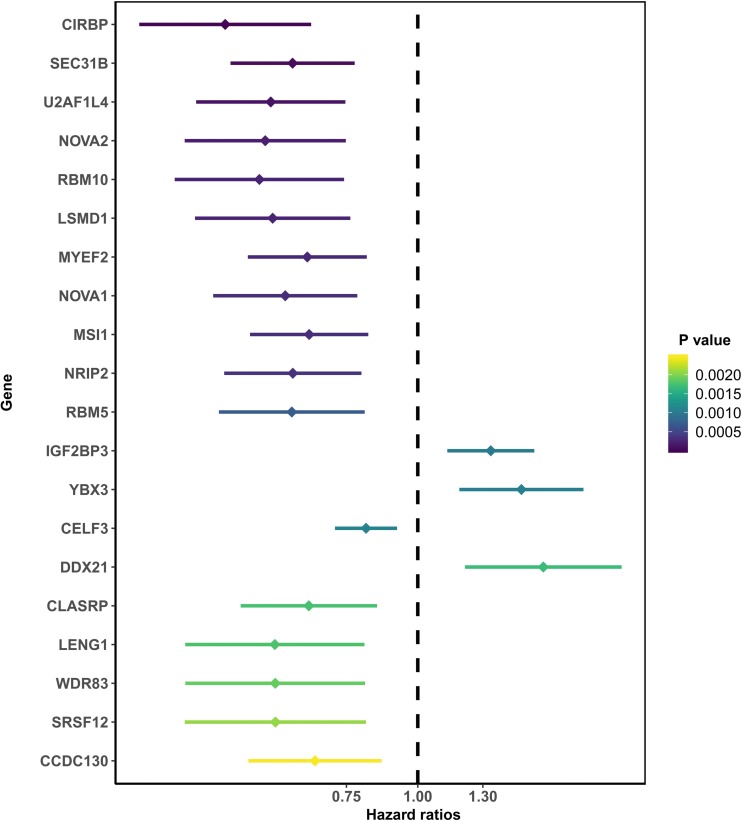
The top 20 most significant survival-associated splicing factors.

After removing those with an OS of less than 90 days, 166 total PAAD patients were included in the present study and were comprised of 90 (54.2%) male and 76 (45.8%) female patients. By integrating 404 SF gene expression profiles and the survival data, we conducted a univariate COX analysis and found 93 SFs genes were correlated with the OS of PAAD patients (*P* < 0.05). The top 20 most significant SFs are listed in Fig. 1.

**Figure 2:**
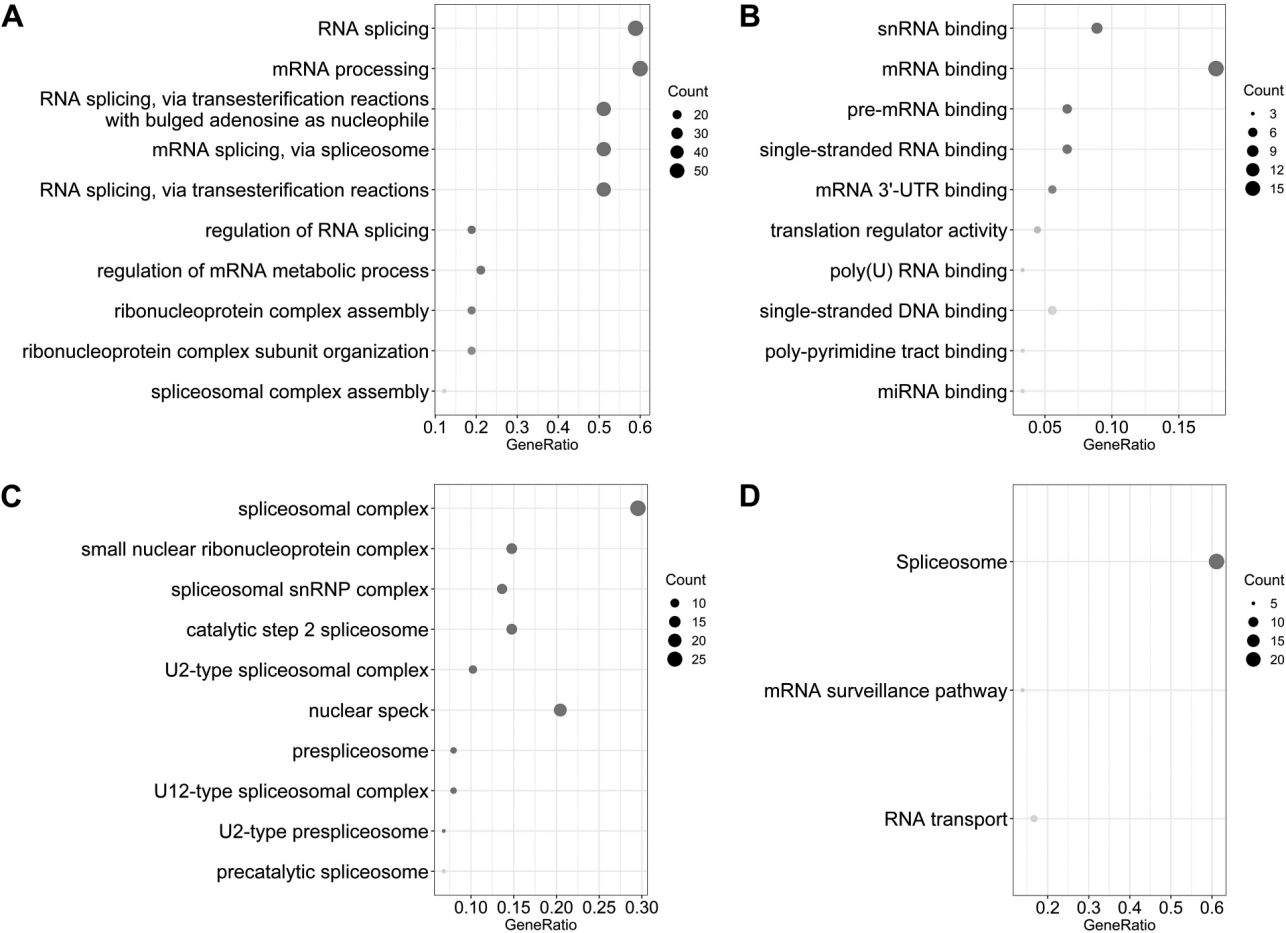
Gene ontology and the Kyoto Encyclopedia of Genes and Genomes (KEGG) pathway analysis of survival associated splicing factors. (A) “RNA splicing,” “mRNA processing,” and “RNA splicing via transesterification reactions with bulged adenosine as a nucleophile” are the three most significant biological process terms. (B) “Spliceosomal complex,” “small nuclear ribonucleoprotein complex,” and “spliceosomal snRNP complex” are the three most significant cellular component terms. (C) The three most significant molecular function terms were “snRNA binding,” “mRNA binding,” and “pre-mRNA binding.” (D) The KEGG pathway analysis revealed that these genes were mainly involved in “spliceosome,” “mRNA surveillance pathway,” and “RNA transport.”

Gene functional enrichment analysis revealed that prognosis-related SFs genes were classified into 61 BPs, 21 CCs, 24 MF, and 3 KEGG pathways. For BPs, the three most significant categories were “RNA splicing,” “mRNA processing,” and “RNA splicing via transesterification reactions with bulged adenosine as nucleophile” (Fig. 2A). For CCs, the three most significant terms were “spliceosomal complex,” “small nuclear ribonucleoprotein complex,” and “spliceosomal snRNP complex” (Fig. 2B). For MF, these genes were mainly involved in “snRNA binding,” “mRNA binding,” and “pre-mRNA binding” (Fig. 2C). Furthermore, we found these SFs genes mainly participated in “spliceosome,” “mRNA surveillance pathway,” and “RNA transport pathways” (Fig. 2D).

### Development of a PI based on SFs

**Figure 3:**
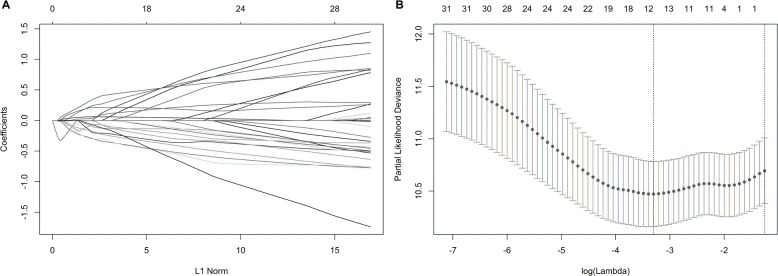
Construction of the prognostic index based on the most significant survival-associated splicing factor genes ( *P* < 0.005) using the LASSO COX regression model. (A) The LASSO coefficient profiles of the splicing factors. A vertical line is drawn at the value chosen by 10-fold cross-validation. (B) Tuning parameter (*λ*) selection cross-validation error curve. The vertical lines were drawn at the optimal values by the minimum criteria and the 1-SE criteria.

**Figure 4:**
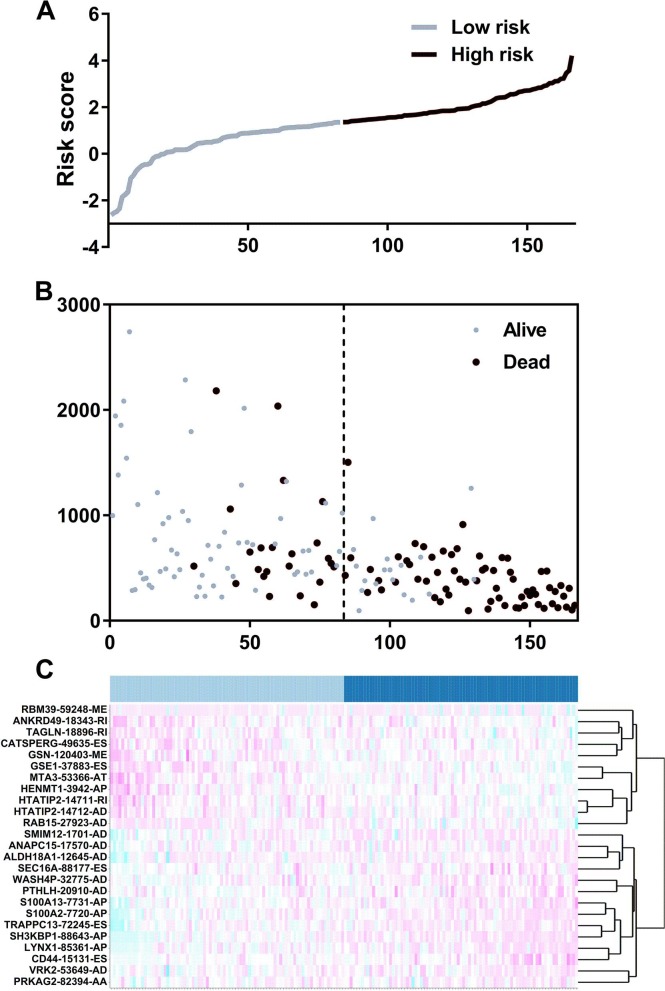
The development of a prognostic index based on splicing factors. (A) Distribution of a risk score by the prognostic signature based on splicing factors. (B) The patients were separated into two groups with distinct survival statuses according to the median value of the risk score. (C) A heatmap shows the expression profiles of the included genes in the high- and low-risk groups.

**Figure 5:**
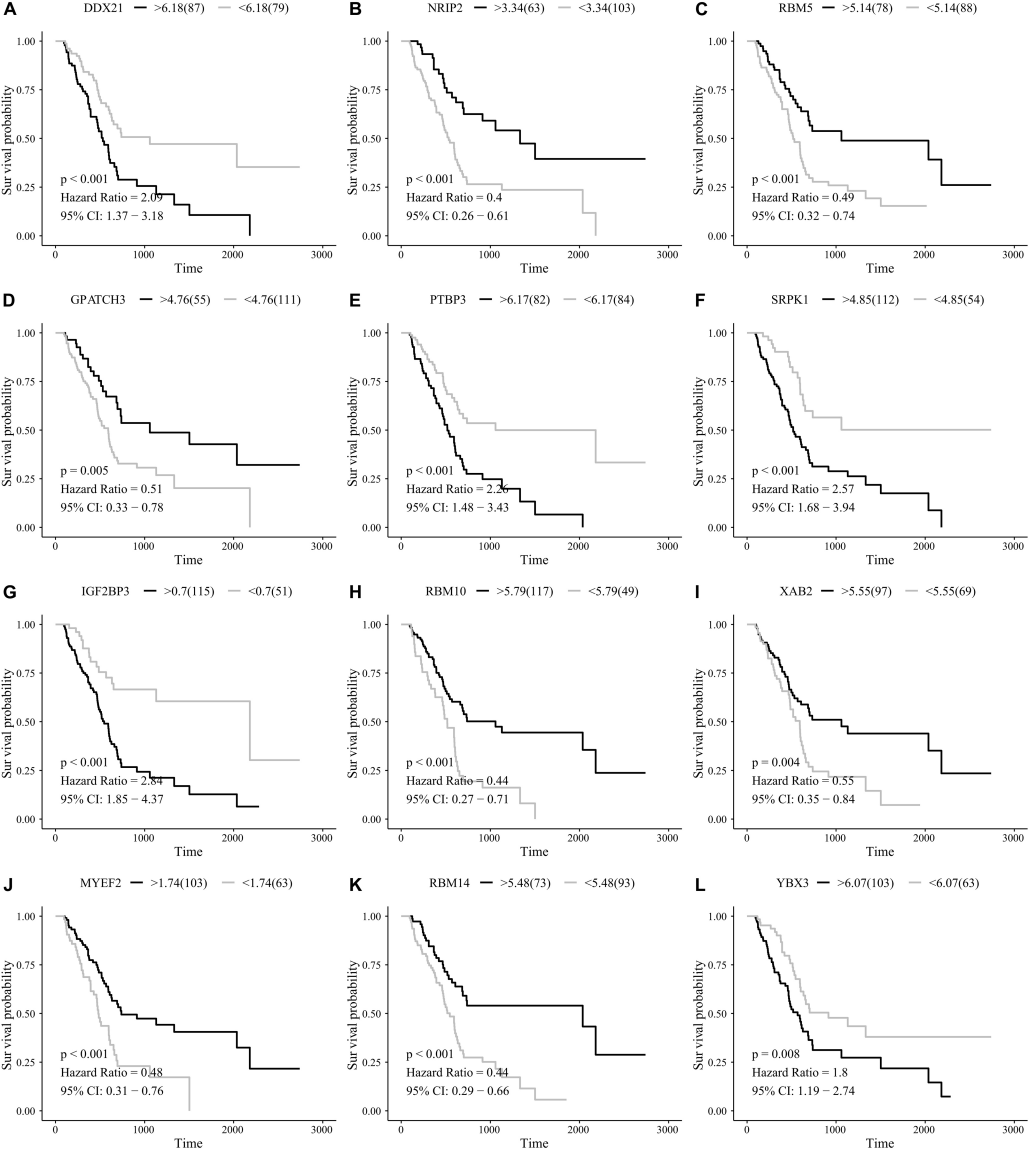
Kaplan–Meier survival plots showed the clinical significance of the splicing factors included in the PI. (A) DDX21, (B) NRIP2, (C) RBM5, (D) GPATCH3, (E) PTBP3, (F) SRPK1, (G) IGF2BP3, (H) RBM10, (I) XAB2, (J) MYEF2, (K) RBM14, and (L) YBX3.

**Figure 6:**
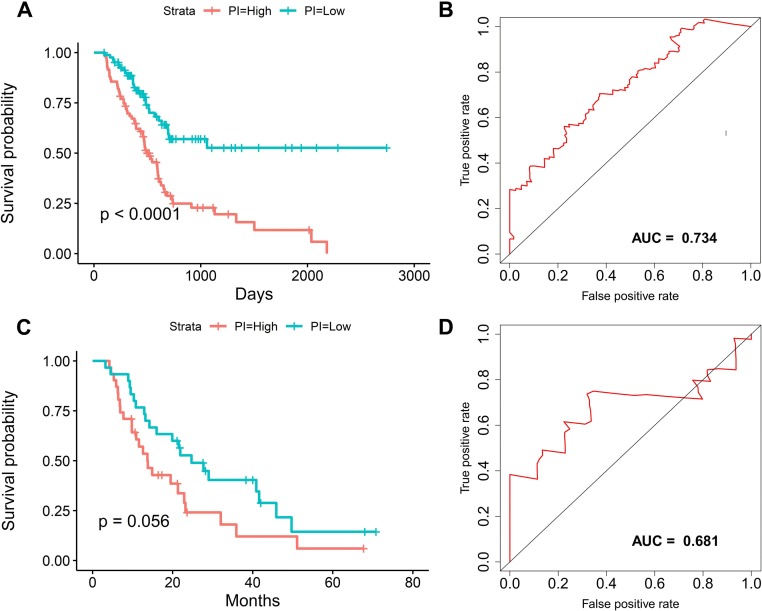
The survival prediction performance of the prognostic index. (A) Kaplan–Meier survival plots suggested that patients in the high-risk group could expect a poor survival. (B) ROC curves with calculated AUCs of prognostic signatures built in the PAAD cohort for risk prediction over 1,500 days.

We suspected that a gene set could exhibit more accurate survival prediction performance than a single gene. Therefore, we constructed an SF-based PI according to the results of the LASSO COX analysis (Fig. 3). This analysis was conducted using the most significant SFs (*P* < 0.005). Finally, 12 SFs were included in the PI, including DDX21, GPATCH3, IGF2BP3, MYEF2, NRIP2, PTBP3, RBM10, RBM14, RBM5, SRPK1, XAB2, and YBX3. The constructed PI based on the 12 SFs = [DDX21 * 0.204800595 + GPATCH3 * (−0.075547356) + IGF2BP3 *0.060551219 + MYEF2 * (−0.16140842) + NRIP2 * (−0.274848438) + PTBP3 *0.217746846 + RBM10 * (−0.096000129) + RBM14 * (−0.147396111) + RBM5 * (−0.289524669) + SRPK1 *0.031528808 + XAB2 * (−0.051783325) + YBX3 * 0.30845434]. Each patient was generated a PI (Fig. 4A). We found that the patients could be separated into two groups with distinct clinical outcomes based on the median PI (Fig. 4B). The heatmap also showed that the included SFs were differentially expressed between the high- and low-risk groups (Fig. 4C). K-M plots were generated to reveal the survival significance of genes included in the prognostic signature (Fig. 5). Based on the SF-based PI median value, PAAD patients could be separated into two groups with distinct clinical outcomes (Fig. 6A). The AUC was 0.734 in 3 year (Fig. 6B). In the validation cohort, patients in high-risk group suffered poorer survival near to statistical significance (Fig. 6C). The AUC was 0.681 in 3 year (Fig. 6D).

### Identification of prognosis-associated AS events

**Figure 7:**
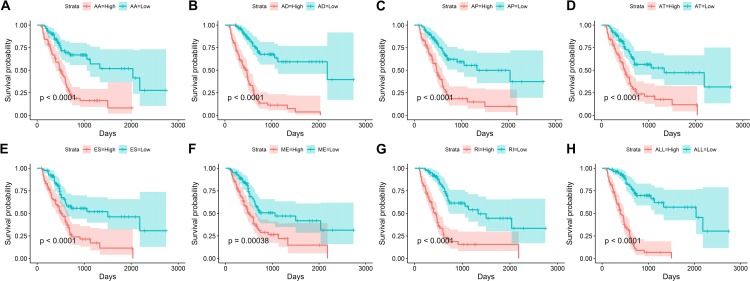
Kaplan–Meier survival plots showed the stratification of the prognostic index based on alternative splicing events. (A) Acceptor Site, (B) Alternate Donor Site, (C) Alternate Promoter, (D) Alternate Terminator, (E) Exon Skip, (F) Mutually Exclusive Exons, (G) Retained Intron, and (H) all types of AS events.

**Figure 8:**
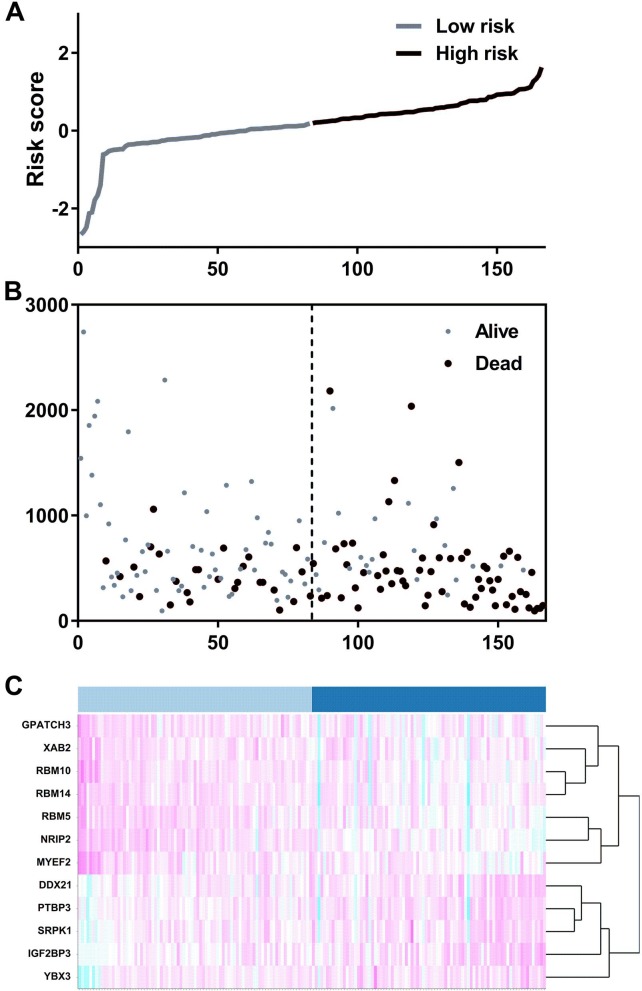
The development of a PI based on alternative splicing events. (A) Distribution of the risk score by the prognostic signature based on alternative splicing events. (B) The patients were separated into two groups with distinct survival statuses by the median value of the risk score. (C) A heatmap displays the expression profiles of the included genes in the high- and low-risk groups.

**Figure 9:**
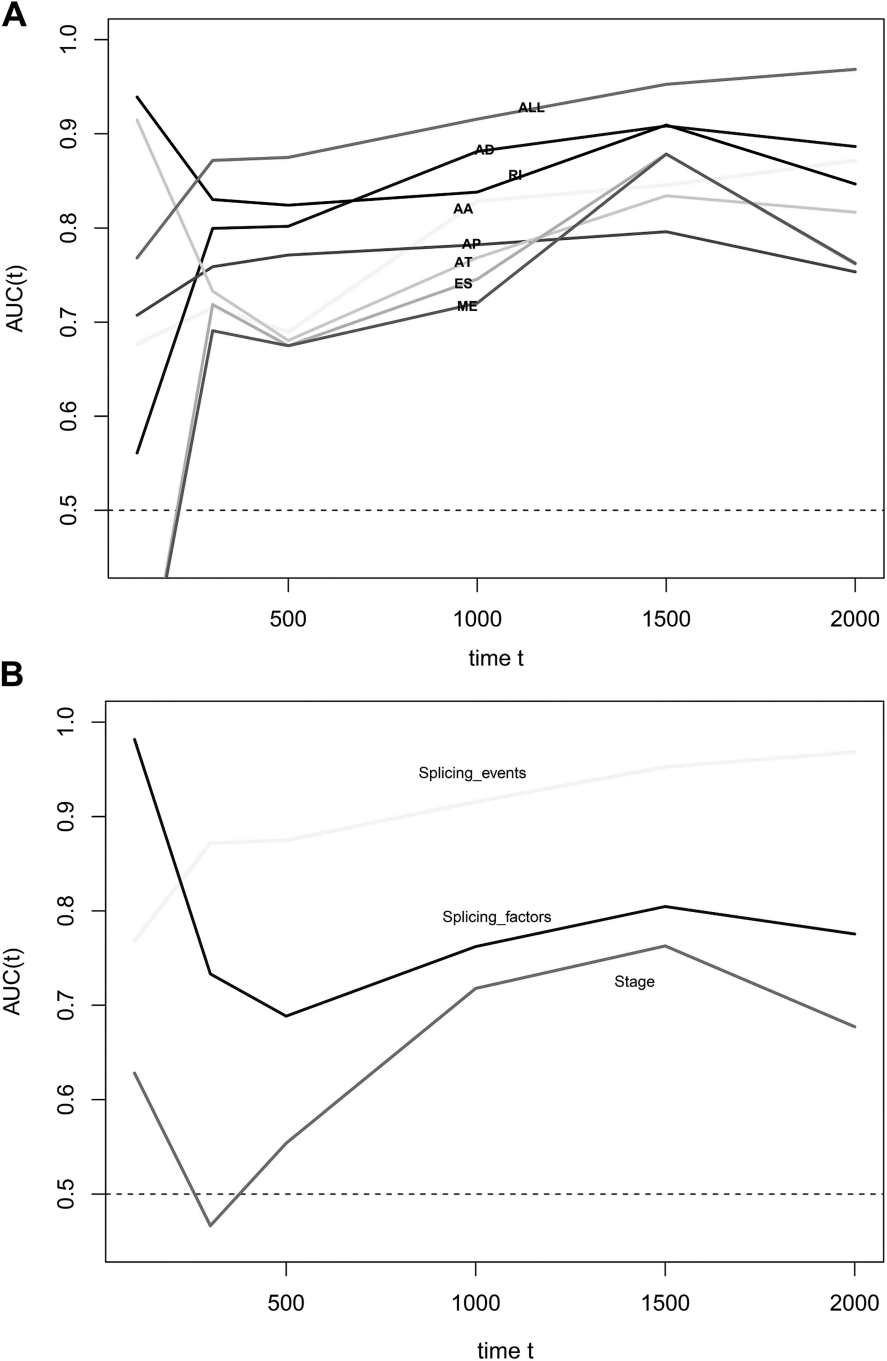
Time-dependent receiver operating characteristic (ROC) curves of the survival prediction systems. (A) ROC curves of prognostic predictors built by one type or all types of alternative splicing events was the highest. (B) The AUC of the ROC at 500, 1,000, 1,500, and 2,000 days, respectively. The AUC of the prognostic index that was based on all types of alternative splicing events was the highest.

We obtained 10,354 AS events for the survival analysis, including 656 AA, 705 AD, 3181 AP, 1394 AT, 3494 ES, 62 ME, and 862 RI. We found that the 26 AA, 35AD, 297 AP, 122 AT, 230 ES, 6 ME, and 70 RI events were most significantly correlated with the OS of PAAD patients (*P* < 0.005). LASSO COX analyses were conducted based on the top 20 most significant OS-associated SFs. Seven PIs based on AA, AD, AP, AT, ES, ME, and RI were finally constructed (Fig. 7). According to the final PI based on AS events, a PI was generated for each patient (Fig. 8A). We found that the patients could be separated into two groups with distinct clinical outcomes based on the median PI (Fig. 8B). The heatmap also showed that the included AS events were differentially expressed between the high- and low-risk groups (Fig. 8C). The time-dependent ROC of PI based on AS events indicated that the final PI possessed the highest AUC (Fig. 9A). The AUCs of SF-based PI, AS-based PI, and TNM are also displayed (Fig. 9B).

### SF-AS regulatory network

**Figure 10:**
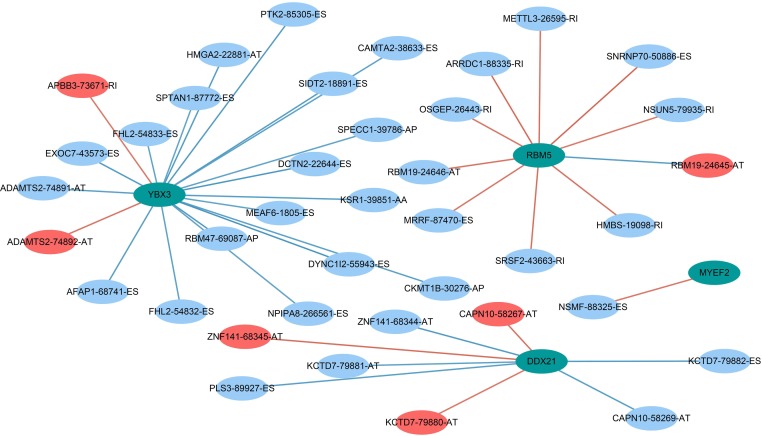
Prognostic splicing factors and the splicing correlation network in PAAD. The construction of an SF-AS regulatory network. Green dots represent the SFs; red dots indicate risky alternative splicing events, and blue dots represent protective events.

AS events are mainly regulated by just a few SFs. Therefore, we decided to explore the prospective regulatory mechanism between SFs and AS events in PAAD. A Pearson correlation analysis was performed and suggested that 33 favorable AS events (blue dots) and 6 risky AS events (red dots) were closely related to the 4 SFs (green dots) (Fig. 10).

## Discussion

We performed a survival analysis focused on the clinical significance of SFs in PAAD based on one of the largest available cancer genomics datasets to develop an excellent prognostic risk score. Although systematic analyses of somatic mutations, copy numbers, gene expression patterns, and associated AS events have been reported (Neelamraju et al., 2018; Sebestyén et al., 2016), many important issues in the field remain unresolved, especially the unique clinical value of SFs in PAAD. Moreover, the AS events related to SFs could also provide novel insight into the molecular function of SFs in PAAD.

PAAD is one of the most lethal cancers and causes a high morbidity. Hence, exploration of the impact of multiple molecular biomarkers is crucial for a prognosis evaluation. Previously, several studies have proposed prognostic signatures for survival prediction. Previously, several studies have proposed prognostic signatures for survival prediction. For example, Yu, Feng & Cang (2018) integrated the miRNA-expression profiles and clinical information of 168 PAAD patients in the TCGA database and developed a two-microRNA signature for the diagnosis and prognosis assessment. Similarly, Shi et al. (2018) proposed a three-lncRNA signature for potential survival prediction, and this signature served as an independent prognostic predictor in PAAD. ROC of the 3-lncRNA signature was 0.716, which is slightly lower than the present study. Researchers have also provided a five-lncRNAs signature that could act as a potential prognostic indicator for PAAD patients by mining the TCGA database (Song et al., 2018). The AUC for the six-lncRNA biomarkers prognostic model was 0.727 at 5 years of OS. These findings provide alternative clinically selectable indicators for PAAD surveillance. The prognostic signature we proposed have well performance when compared with previous molecular index. However, the prognostic signatures proposed based on the global alterations of genes could only provide limited information. In the present study, we proposed a risk score that was mainly focused on the expression profiles of SFs in PAAD. Because the roles of SFs in PAAD have not been fully explored, more studies are needed to reveal their clinical significance. New findings about the relationships between SFs and AS events could offer a broader insight into the molecular process of PAAD.

We finally proposed a prognostic signature based on 12 SFs. Interestingly, some of these 12 SFs have been reported in PAAD. Schaeffer et al. (2010) concluded that IGF2BP3 was upregulated in PAAD, and its overexpression indicated poor survival based upon an immunohistochemical analysis of 127 PAAD patients. This result was consistent with our findings. Subsequent analyses revealed that IGF2BP3 could promote cell invasiveness and the metastasis of pancreatic cancer (Taniuchi et al., 2014). RBM5 has also been proven to be downregulated in pancreatic cancer, and reduced RBM5 expression has a close association with poor clinicopathological features (Peng et al., 2013). Furthermore, Hayes et al. (2006) reported that knockdown of the expression levels of SRPK1 in pancreatic tumor cells could decrease the proliferative capacity and increase the apoptotic potential of pancreatic tumor cells. These results suggest that SRPK1 could be an effective therapeutic target for pancreatic cancer. Previous reports about the SFs we proposed provide some evidence about their crucial functions. Although previous studies have mentioned their clinical significance and molecular function, a comprehensive exploration is still needed.

The prognosis anticipating value of AS events was widely explored recently for its limitless potential for clinical applications. Several studies have attempted to investigate the prognostic value of AS events in several types of cancer. For example, some researchers have explored the prognostic value of AS events in lung cancer (Li et al., 2017). This groundbreaking research pointed out the well-known value of AS events. Subsequently, researchers found that AS exhibited an effective prognosis prediction value in thyroid cancer (Lin et al., 2019), gastrointestinal pan-adenocarcinomas (Lin et al., 2018), and diffuse large B-cell lymphoma (Zhang et al., 2018). Algorithmically, the established prognostic models were based on the PSI value. This value is a useful method for quantifying AS events and demonstrating its clinical value. To the best of our knowledge, we are the first group to integrate the clinical parameters and PSI values of AS events. In this study, we also constructed an SF-AS potential regulatory network, which provides the underlying mechanisms of SFs in PAAD. Indeed, many survival associated splicing events has been validated. For example, previous study has reported that VEGFA/76336/ES significantly associated poor survival in pancreatic cancer (Zhang et al., 2017). Furthermore, many SFs have been validated important in splicing regulation and regulate the processes of tumors. RBM5 could promote exon 4 skipping of AID pre-mRNA (Jin et al., 2012). YBX3 was found to be related to spliceosomes in large-scale spliceosome capture and mass spectrometry analyses (Rappsilber et al., 2002).

As the present study was based on an *in silico* analysis, there are several inevitable limitations that should be mentioned. First, no other independent study, especially a prospective study, has validated the prognostic signatures we proposed. Second, the clinical parameters related to the prognosis of PAAD have not been fully investigated.

## Conclusions

In conclusion, we systematically explored the clinical significance of SFs in PAAD patients. More importantly, a prognostic signature based on the prognosis-associated SFs was constructed to separate PAAD patients into two groups with distinct clinical outcomes. These findings could provide more information about the clinical value of SFs. The SF-AS regulatory network provided information regarding the molecular functions of SFs.

##  Supplemental Information

10.7717/peerj.8380/supp-1 File S1 R Code used for analysis

10.7717/peerj.8380/supp-2 File S2 Raw data

10.7717/peerj.8380/supp-3 File S3 The raw data of results of univariate Cox for splicing events

10.7717/peerj.8380/supp-4 File S4 The raw data of results of univariate Cox for splicing factors

## Additional Information and Declarations

### Data Availability

The following information was supplied regarding data availability: The raw data of gene symbol, R codes, results of univariate Cox for splicing events and splicing factors are available in the Supplementary Files.
